# Femtosecond laser combined with non-chopping rotation phacoemulsification technique for soft-nucleus cataract surgery: a prospective study

**DOI:** 10.1038/srep18684

**Published:** 2016-01-05

**Authors:** Hui Chen, Haotian Lin, Wan Chen, Bo Zhang, Wu Xiang, Jing Li, Weirong Chen, Yizhi Liu

**Affiliations:** 1 State Key Laboratory of Ophthalmology, Zhongshan Ophthalmic Center, Sun Yat-sen University, Guangzhou, Guangdong, 510060, China

## Abstract

Soft-lens cataract surgeries are becoming increasingly common for cataract surgeons and chopping the soft nucleus using conventional techniques is problematic. We introduced a femtosecond laser combined with a non-chopping rotation phacoemulsification technique for soft-nucleus cataract surgery and evaluated the safety and efficacy of using this technique. Sixty-six patients with soft-nucleus cataracts ranging from grade 1~3 were divided into 3 groups based on nuclear staging. Those groups were further divided into three subgroups: femtosecond laser pretreatment combined with a non-chopping rotation phacoemulsification technique (subgroup 1), conventional manual cataract surgery with a non-chopping rotation technique (subgroup 2) and conventional manual cataract surgery with a quick-chop technique (subgroup 3).Patients were followed up at 1, 7, and 30 days after surgery. There was an 84.6% and a 63.34% reduction in ultrasound time and cumulative dissipated energy, respectively, between the subgroup 1 and the subgroup 3; and this was associated with a 36.1% and 29.7% reduction in endothelial cell loss and aqueous flare. There were no adverse events at the follow-up times. With its reduced ultrasound energy, endothelial cell loss and aqueous flare, the femtosecond laser pretreatment combined with a non-chopping rotation technique was more efficient than conventional manual cataract surgery for soft-nucleus cataracts.

With cataract surgery becoming the most common ophthalmic surgical procedure worldwide^1^, cataract surgery is now available to an increasing number of people. Patients no longer need to suffer hyper-mature or mature cataracts and can undergo cataract surgery early enough to prevent advancement to these states. Therefore, for cataract surgeons, soft-lens cataract surgeries are becoming increasingly common. However, the fragmentation of soft-lens cataracts may pose a potential challenge for cataract surgeries because chopping the soft nucleus using conventional techniques may not only waste phacoemulsification power but also increase the risk of chop-related complications^2^. This fact has rekindled the interest in non-chop techniques^3^. We have introduced a non-chopping rotation and axial rotation phacoemulsification technique for the removal of soft-lens cataracts. A previous study showed that for grade 1 and 2 nuclei, non-chopping rotation and axial rotation techniques outperformed the quick-chop technique in ultrasound time (UST), cumulative dissipated energy (CDE), corrected distance visual acuity (CDVA), central corneal thickness (CCT), and endothelial cell loss (ECL)^4^.

Recently, a new generation of femtosecond lasers was introduced for cataract surgery that can perform the key steps of the cataract procedure, including lens fragmentation^5^,^6^,^7^. The advantages of the use of a femtosecond laser in cataract surgery have been shown in several studies and include a reduction in ultrasound phacoemulsification energy after lens softening^8^,^9^,^10^,^11^. Several lens fragmentation patterns, including a cross pattern, cake pattern, grid pattern, cylindrical pattern and a hybrid pattern, have been described for the femtosecond laser system. Liquefaction by the cylindrical pattern has been recommended for soft nuclei. Inspired by the femtosecond laser cylindrical pattern procedure for lens softening, we combined the non-chopping rotation technique with the femtosecond laser cylindrical pattern to develop our own technique for removal of soft nuclei, to minimize the necessary phacoemulsification energy and time. Previous studies have shown that the level of ultrasound use influences anterior chamber flare values, which is a sign of an increased permeability of the blood–aqueous barrier^12^. With the introduction of femtosecond laser pretreatment, there is the need to evaluate the safety profile of this new technology with respect to its potential to alter postoperative inflammation. The aim of this paper is to evaluate the safety and efficacy of femtosecond pre-treatment with a non-chopping rotation technique in soft nuclei.

## Results

Sixty-six cataract patients were enrolled in the study. Of these, 22 were assigned to the femto + non-chopping rotation group (subgroup 1), 22 were assigned to the manual + non-chopping rotation group (subgroup 2), and 22 were assigned to the manual + quick-chop group (subgroup 3). All patients completed the study. Patient demographics and baseline characteristics were similar among 3 subgroups. The mean age was 65.27 ± 11.45 years in subgroup 1 versus 65.64 ± 7.16 years in subgroup 2 and 66.86 ± 11.09 in subgroup 3 (P > 0.05). The mean cataract grade was 2.27 ± 0.63 in subgroup 1 compared with 2.18 ± 0.66 in subgroup 2 and 2.14 ± 0.64 in subgroup 3. Cataract grades among the subgroups analyzed were not significantly different (P > 0.05). There were 12 women (54.5%) in subgroup 1 compared with 11 women (50.0%) in subgroup 2 and 15 women (68.2%) in subgroup 3 (P > 0.05). No patient developed anterior capsule tears, posterior capsule ruptures, zonular dehiscence, vitreous prolapse or loss, phacoemulsification burns, or phacoemulsification bites. No adverse intra- or postoperative events were observed in any patient during the follow-up period.

**Figure 1:**
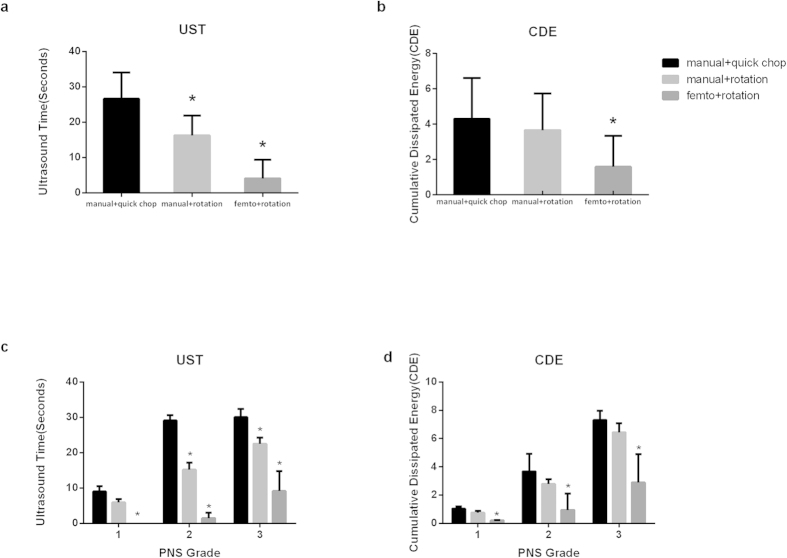
(**a**)Ultrasound time (UST) comparison among the three subgroups: A significant reduction in time in the femtosecond laser pretreatment with non-chopping rotation group (femto + rotation). **(b)** Cumulative dissipated energy (CDE) comparison among three subgroups: A significant reduction in energy was observed in the femtosecond laser pretreatment with non-chopping rotation group. **(c)** Mean ultrasound time in PNS (Pentacam automated nucleus) stages. **(d)** Mean cumulative dissipated energy in PNS stages. *Significant difference when compared with the manual quick chop group (manual + quick chop); P < 0.01.

The mean UST and CDE were significantly lower in the laser group than in other two manual groups (P < 0.001) for each nucleus density grade (Fig. 1a,b). The differences in UST and CDE between subgroups according to nucleus hardness were statistically significant. The UST and CDE consistently increased in eyes with higher grades of nucleus density (Fig. 1c,d).

The Mean UST was significantly lower in subgroup 2 compared with subgroup 3. The overall reduction in UST between groups was 38.99% (16.24 ± 5.65 vs. 26.62 ± 7.38, P < 0.0001; Fig. 1a). This reduction was significant for all cataract grades. A significant improvement was observed in the laser group after using the cylindrical lens fragmentation method while maintaining the same non-chopping rotation phacoemulsification technique (4.10 ± 5.21 vs. 16.24 ± 5.65, P < 0.0001). This represents another 74.75% reduction in mean UST. In other words, the overall reduction in mean UST was 84.6% compared with subgroup 3(P < 0.0001). There were 10 cases (45.5%) in the laser group with 0 UST compared to no cases in the manual groups (P < 0.0001).

Mean CDE was reduced by 15.08% in subgroup 2 compared to subgroup 3 (3.66 ± 2.07 vs.4.31 ± 2.31, P = 0.400; Fig. 1b). Mean CDE also improved 56.83% when assisted by femtosecond laser pretreatment (1.58 ± 1.77 versus 3.66 ± 2.07, P = 0.011). This represents a further 63.34% reduction in mean CDE (P < 0.0001).

**Figure 2:**
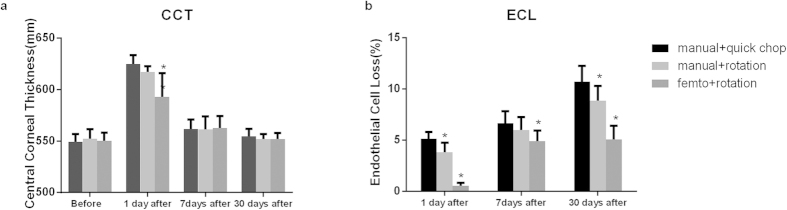
(**a**) Central corneal thickness (CCT) and (**b**)endothelial cell loss (ECL) over time. *Significant difference when compared with the manual quick chop group; P < 0.01.

The average CDVA on postoperative days 1 was significantly better in subgroup 1 than in subgroup 2 (P = 0.002) and subgroup 3 (P < 0.001). The increase in CCT compared to baseline was significantly reduced in laser group at postoperative days 1 (P < 0.001) and 7 (P < 0.001) (Fig. 2a). At postoperative day 1, day 7 and day 30, significantly less ECL was observed in subgroup 1 than in subgroup 2 (all P < 0.0001) and subgroup 3 (all P < 0.0001) (Fig. 2b). There were no statistically significant differences in CDVA or CCT among the subgroups at day 30 after operation (both P > 0.05) (Table 1).

**Figure 3:**
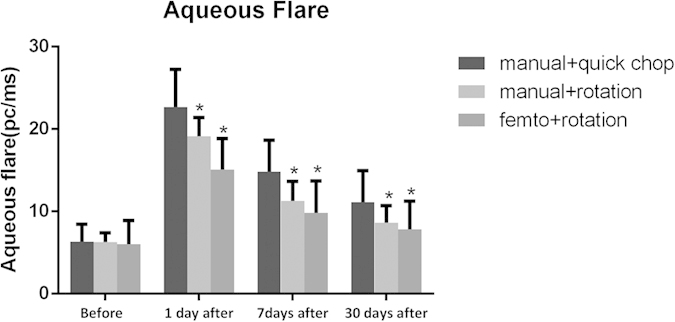
Aqueous flare over time. *Significant difference when compared with the manual quick chop group; P < 0.01.

The laser flare values were significantly lower in subgroup 1 than in subgroup 2 (15.05 ± 3.82 vs. 19.14±2.24, P = 0.001) and subgroup 3 (15.05 ± 3.82 vs. 22.67 ± 4.60, P < 0.001) at postoperative day 1 (Fig. 3). One weeks after surgery, value decreased to 9.80 ± 3.91 pc/ms in subgroup 1, compared to 11.29 ± 2.36 pc/ms in subgroup 2 (P = 0.408) and 14.81 ± 3.33 pc/ms in subgroup 3 (P < 0.001) (Fig. 3). At the 30 days postoperative visit, laser flare value was 7.82 ± 3.44 pc/ms in subgroup 1, compared to 8.65 ± 2.06 pc/ms in subgroup 2 (P = 1.000) and 11.13 ± 3.32 pc/ms in subgroup 3(P = 0.002)(Fig. 3).

## Discussion

In our study, we compared the safety and efficacy of surgical techniques in 66 eyes in 66 patients with soft-nucleus cataracts. Of these, 22 patients had femtosecond laser pretreatment with the non-chopping rotation technique (study group), whereas standard cataract surgery followed by phacoemulsification with or without non-chopping rotation techniques was performed on the other patients (control groups). The results showed that phacoemulsification time and energy can be significantly reduced by using the femtosecond laser-assisted technique described here. Furthermore, postoperative corneal edema and endothelial cell loss can be effectively reduced. Laser flare meter measurements showed that femtosecond pretreatment combined with non-chopping rotation technique also resulted in less aqueous flare than standard manual cataract surgery.

To eliminate phacoemulsification damage, changes in surgical techniques and equipment have been introduced to cataract surgeries. For soft-nucleus cataracts, various chopping techniques such as quick chop technique performed without previous sculpting have evolved from the standard divide-and-conquer technique to achieve minimal ultrasound power and to avoid surgically-induced trauma^3^,^13^,^14^. In our previous study, we introduced modified techniques named “axial rotation” and “non-chopping rotation technique” for the removal of soft- to medium-hard-nucleus cataracts and found these techniques to be more effective than the quick-chop technique, as shown by intraoperative UST, CDE, and the postoperative corneal trauma index^4^. However, with higher expectations for a rapid visual recovery, the goal of zero phacoemulsification time remains a challenge for cataract surgeons. Recently, the introduction of femtosecond laser-assisted cataract surgery with lens pre-fragmentation has introduced the possibility of a further reduction in phacoemulsification energy. Inspired by this technological advance, we combined a cylinder segmentation pattern of femtosecond laser pretreatment with a non-chopping rotation technique for soft-lens cataract surgery.

First, refinements in lens fragmentation patterns have allowed surgeons to eliminate phacoemulsification in patients pretreated with a femtosecond laser. The cylindrical pattern is used to soften the nucleus by dividing the lens into concentric rings that arise from the back of the cataratous lens and curve toward the anterior lens. After pre-fragmentation of the lens using a cylindrical pattern, which provides a clear tract mark for the lens rotation controlled by the phacoemulsification tip, the lens can be removed easily with great reliability. When combined with a non-chopping technique during phacoemulsification, as the nucleus rotates around the axis following the pre-exist tract mark, its volume could easily diminishes along the cylindrical pattern from the nucleus margin circle to the center. Thus, the pre-fragmentation by femtosecond laser ensures the continual and smooth rotation of the nucleus and further facilitates the emulsification procedure, thereby minimizing the necessary phacoemulsification energy and time.

Another advantage of femtosecond laser-assisted cataract surgery is that a pre-laser treatment may further reduce phacoemulsification energy and time through the automation of corneal incisions and anterior capsulotomy^15^,^16^. Previous studies have found that femtosecond laser assistance significantly reduced both the effective phacoemulsification time (EPT) and mean phacoemulsification energy. Palanker *et al.* show a 40% reduction in US energy compared with manual cataract surgery^17^. Abell et al. report a 70% reduction in mean EPT compared with the traditional technique^9^. Mayer et al. also demonstrate a significantly reduced EPT, at 1.58 ± 1.02 seconds compared to 4.17 ± 2.06 seconds in the manual group (P < 0.001), which correlates positively with preoperative lens opacity^10^. Conrad Hengerer et al. find a significant reduction (96%) in EPT that was influenced by differences in fragmentation patterns^8^,^18^.

The results of this study should be interpreted according to its strengths and limitations. First, our study showed that further reduction of phacoemulsification energy and time has been achieved by combing femtosecond laser pretreatment and a non-chopping rotation technique. There was a near elimination of the need for phacoemulsification for grade 1 to grade 3 nuclei. It has been shown that lower phacoemulsification energy during cataract surgery may increase the safety of the surgery with respect to postoperative corneal swelling and loss of endothelial cells^19^,^20^,^21^. Consequently, reducing the energy delivered to the eye may be associated with earlier improvements in postoperative visual acuity, along with reduced endothelial cell loss and corneal edema^22^. This was confirmed in our study, in which visual acuity 1 day after surgery was significantly better in the study group than in the control groups^23^. A significant reduction in the mean CCT and corneal endothelial cell loss was observed in the femtosecond pretreatment group.

Furthermore, consistent with previous reports, our study showed that anterior segment inflammation was reduced after femtosecond laser pretreatment combined with non-chopping technique compared to the conventional manual cataract surgery, and this appeared to be due to a reduction in phacoemulsification energy^24^,^25^. Postoperative inflammation is associated with a breakdown of the blood–aqueous barrier which is generally manifests as mild iritis, increased cells and protein in the anterior chamber, and macular edema. The occurrence of postoperative inflammation would hinder the visual recovery and induce associated pain, which is a challenge for both surgeons and patients. The introduction of femtosecond pretreatment has led to further reductions in phacoemulsification energy requirements as we mentioned above. Combined with non-chopping rotation technique, this automated surgical technique could further reduce surgical trauma and the resulting inflammation. Our study suggested that this new technique for soft-nucleus cataracts is advantageous especially for those patients who have high expectations for refractive surgery, because it decreases postoperative inflammation, increases patients’ ´satisfaction, and reduces the risk for endophthalmitis.

Further clinical studies may focus on custom fragmentation techniques and various softening patterns of use in femtosecond laser pretreatments, in the hopes of achieving further reductions in the ultrasound energy required. This new surgery technique permits a certain degree of automation and renders surgical success less dependent on the surgeon. In the near future, further developments in technology and surgical techniques may make automated cataract extraction possible. This study’s limitations include both its relatively short-term follow-up and its small sample size. Because the potential recovery time for endothelial cells after cataract surgery lasts up to 6 months, longer-term observation is necessary and a much larger sample size would be required. In summary, a significant reduction in phacoemulsification energy and time is made possible through the use of the femtosecond laser pretreatment when combined with a non-chopping technique for soft-nucleus cataract surgery. The goal of 0 CDE could be achieved using a combination of improved surgical techniques and lens fragmentation methods and parameters, which may not only lead to decreased corneal endothelial cell loss and corneal edema during the early postoperative phase but also result in faster visual recovery.

## Methods

### Patients

**Figure 4:**
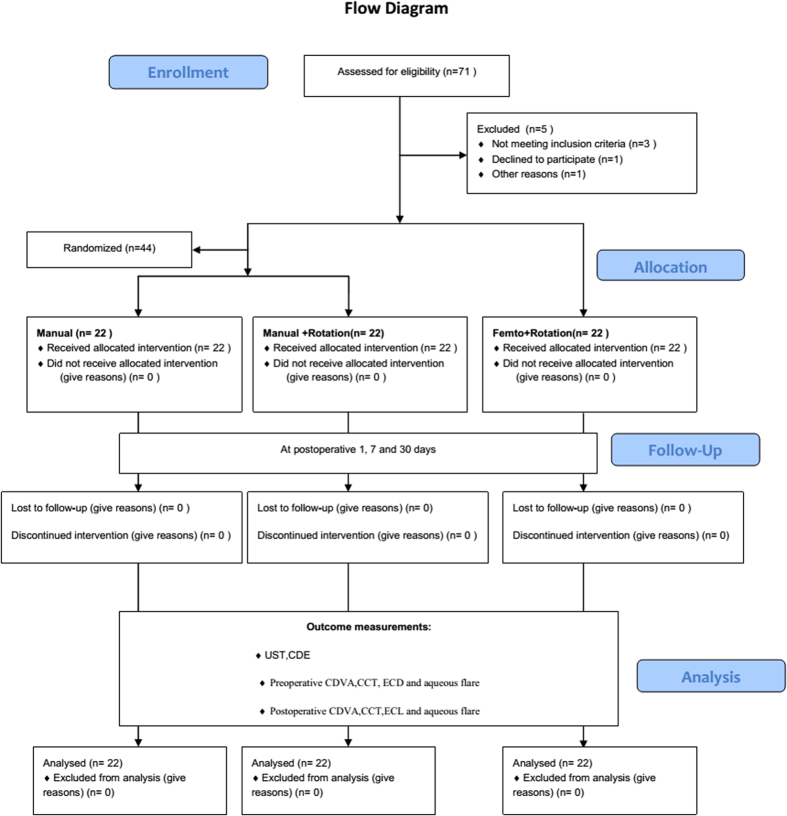
Summary and flow diagram of the trial design (n = number of eyes). UST = Ultrasound time; CDE = Cumulative dissipated energy; CCT = central corneal thickness; CDVA = corrected distance visual acuity; ECD = endothelial cell density; ECL = endothelial cell loss.

This was a prospective, consecutive, investigator-masked, nonrandomized, cohort study that was performed at a single center (Fig. 4). The study was approved by the Human Research Ethics Committee of the Zhongshan Ophthalmic Center and was performed in accordance with the Declaration of Helsinki. Written informed consent was obtained from all patients. Consecutive patients with cataract nuclei graded 1~3 who planned to have either femtosecond laser-assisted cataract surgery or manual cataract surgery with insertion of a posterior chamber intraocular lens (IOL) were enrolled in the study. The Pentacam HR Scheimpflug imaging system was used for cataract grading (Pentacam nucleus staging [PNS]) (Pentacam; Oculus, Wetzlar, Germany)^26^,^27^. An automatic alignment of the Pentacam nucleus staging between grades 0 and 5 was performed in all preoperative cases after the administration of mydriatic eye drops (tropicamide 0.5% and phenylephrine 5%) (Santen Pharmaceutical, Osaka, Japan). Distinctive images acquired for the right eye (90°–270°) and for the left eye (270°–90°) were analyzed.

All of the patients were given the option of having femtosecond laser–assisted cataract surgery. Patients who elected to have femtosecond laser–assisted cataract surgery were placed in the laser group (subgroup 1: femtosecond laser pretreatment combined with non-chopping rotation phacoemulsification techniques), and the remaining patients were randomly placed (using computer randomization) into the manual groups (subgroup 2: conventional manual cataract surgery with non-chopping rotation techniques and subgroup 3: conventional manual cataract surgery with quick chop techniques). Exclusion criteria included an undilated pupil of 7.0 mm or larger, endothelial cell density (ECD) of less than 1500 cells/mm2, and other visual or systemic disorders that might affect vision (e.g., diabetic retinopathy, glaucoma, age-related macular degeneration, uveitis, previous intraocular surgery or trauma history). Based on our preliminary data, at least 22 patients needed to be included in the analysis to achieve sufficient power in the statistical calculations. The 22 cases in the study group were paired 1:1 with patients from a database of 50 consecutive cases who underwent conventional cataract surgery according to age, sex and PNS. A summary of the enrollment and measure process is shown in Figure 1.

### Surgical Technique

All surgeries were performed at the Zhongshan Ophthalmic Center (Guangzhou, China). All manual and femtosecond laser surgeries were performed by the same experienced surgeon (Yizhi Liu). According to standard cataract surgery protocols, patients administered levofloxacin 0.5% (Quixin; Santen, Napa, CA) into the operated eye 4 times daily for 3 days before surgery. All of the surgeries were performed under topical anesthesia with 0.5% proparacaine hydrochloride (0.5% alcaine, Alcon-Couvreur, Belgium). Pupillary dilation was achieved before surgery with 1% tropicamide, 10% phenylephrine, and 1% cyclopentolate. In patients who had manual cataract surgery, 2.8 mm clear corneal and side-port incisions were made with a disposable keratome (Alcon Laboratories, Fort Worth, TX, USA). Continuous curvilinear capsulorhexis was performed with a cystotome and a capsular forceps. Lens segmentation was completed with the standard quick-chop technique in subgroup 3 and the non-chopping rotation technique, as described previously, in subgroup 2 4.

**Figure 5:**
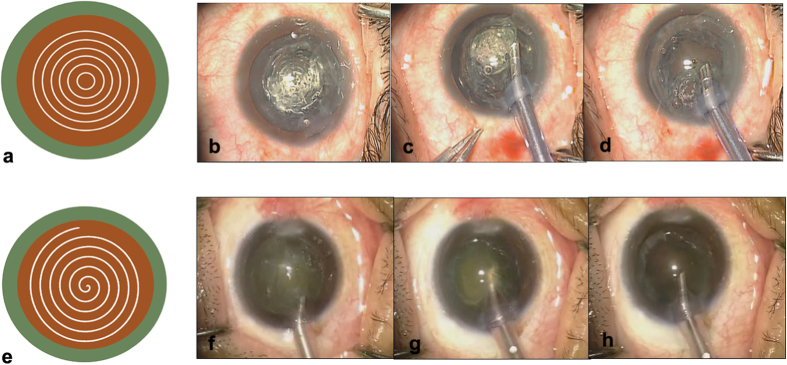
(**a**)Diagrams showing cylindrical lens-softening pattern (6 central chop cylinders with a diameter of 2 mm). **(b–d)** Intraoperative photographs showing phacoemulsification of the nucleus using femtosecond laser pre-fragmentation with a non-chopping rotation technique. The phacoemulsification tip holds the nucleus margin; the nucleus begins to rotate around and is emulsified gradually along a cylindrical pattern from the nuclear margin circle to the center. (**e**) Diagrams showing the rotating tract of nucleus and the phacoemulsification tip gradually devouring the nucleus from margin circle to the center when using a non-chopping rotation technique with manual phacoemulsification. **(f–h)** Intraoperative photographs showing that the phacoemulsification tip holds the nucleus margin; the nucleus begins to rotate and is gradually emulsified.

In subgroup 1, femtosecond laser-assisted cataract surgery was performed using the LenSx femtosecond laser (Alcon Laboratories, Fort Worth, TX, USA). Corneal applanation was performed using a SoftFit interface (Alcon Laboratories, Fort Worth, TX, USA). After manual verification of each procedural step, laser treatment was performed. Primary corneal incisions were placed every 2.8 mm and the attempted capsulotomy diameter was 5.0 mm in all cases. A cylindrical lens-softening pattern (6 central chop cylinders with a diameter of 2 mm) was used (Fig. 5a). The laser pulse energy was set to 7 mJ for corneal incisions and 5 mJ for capsulotomy and lens fragmentation. Following the laser procedure, the patient was transferred to the operating room for the remaining standard phacoemulsification procedure.

For all of the patients, the phacoemulsification procedure was performed using an OZil Torsional Infiniti phacoemulsification system with an OZil torsional handpiece and a 0.9-mm Kelman ABS 45° MicroTip (Alcon Laboratories, Fort Worth, TX, USA) with a 0.9-mm Ultra MicroInfusion Sleeve (Alcon Laboratories, Fort Worth, TX, USA). Phacoemulsification settings were uniform for all patients as follows: torsional continual mode with 100% amplitude and vacuum setting at 400 mm Hg in Custom 1 (for nuclear removal) and 200 mm Hg in Custom 2 (for epinuclear removal). In subgroup 1, the supranuclear cortex was removed by aspiration using the phaco tip. The softened nucleus was aspirated by using a non-chopping rotation technique, with or without US phacoemulsification energy. The phacoemulsification tip was placed beside the capsulorhexis edge, in contact with the nuclear–epinuclear interface. The lens was held firmly onto the phacoemulsification tip by linear aspiration with the nucleus margin held slightly outside the capsular bag. The nucleus was rotated when phacoemulsification power was applied, and its volume diminished along the laser pretreatment tract from the nucleus margin circle to the center (Fig.5b–d). According to the degree of hardness, the phacoemulsification energy was linearly controlled by a foot pedal (depth in position three) to phacoemulsify the nucleus gradually. In subgroup 2, a non-chopping rotation technique was used without the tract made by femtosecond laser pre-fragmentation (Fig. 5e~h). Residual cortex removal and posterior capsule polishing were performed using a bimanual I/A system through the nasal and temporal incisions. After the successful removal of the lens cortex, all of the cohorts received IOL (AcrySof SN60WF, Alcon Laboratories, Fort Worth, TX, USA) implantation in the capsular bag. All of the femtosecond laser-assisted and phacoemulsification procedures and IOL implantations were performed by the same surgeons, who were experienced in all three of the phacoemulsification techniques used in the study.

### Outcome Measures

The main outcome parameters were UST and mean CDE. The UST represented the duration that the foot pedal remained in the third position. The mean CDE power indicated the average percentage of power spent during the ultrasound time. The CDE was calculated as follows: torsional cumulative dissipated energy = average torsional amplitude × torsional time × 0.4. All of the CDE values were recorded and displayed on the screen of the phacoemulsification system.

Safety endpoints were based on adverse events such as incomplete capsulotomy, anterior capsule tears, posterior capsule ruptures, IOL malposition, and iris damage. Postoperative evaluations were performed by another ophthalmologist who did not participate in the surgery and was masked to the treatment assignment. Patients were examined 1, 7, and 30 days after surgery. Postoperative CDVA and complications were both documented. Central corneal thickness was measured with the Anterior Segment Imaging Visante OCT 1000 (Zeiss Meditec, Dublin, CA). The endothelial cell count was measured using a noncontact specular microscope (SP-2000 P; Topcon Corporation, Tokyo, Japan). Aqueous flare was measured with a laser flare meter (FC-2000, Kowa, Tokyo, Japan).

### Statistical Analysis

The SPSS software package (version 11.5, SPSS, Inc., Chicago, IL, USA) was used for statistical analysis. For all groups, the differences in age, UST and CDE among 3 subgroups were tested by one way analysis of variance (ANOVA). The preoperative nuclear density and sex difference among 3 subgroups were analyzed using the chi-square test. In each group, repeated-measures analysis of variance was used to analyze parameters in 3 subgroups. If the results were statistically significant, the data were compared between each subgroup. The Fisher least-significant-difference test was used to calculate the level of significance of differences among the data in a spherical distribution. Bonferroni modification of the t-test was applied to compare data that was not in a spherical distribution. A P value < 0.05 was considered statistically significant in all tests except for Bonferroni modification of the t test, for which P < 0.015 was considered statistically significant in the comparison of the 3 subgroups.

## Additional Information

**How to cite this article**: Chen, H. *et al.* Femtosecond laser combined with non-chopping rotation phacoemulsification technique for soft-nucleus cataract surgery: a prospective study. *Sci. Rep.* **6**, 18684; doi: 10.1038/srep18684 (2016).

## Figures and Tables

**Table 1:** A comparison of preoperative CCT,ECD and aqueous flare and postoperative CDVA, CCT, ECL and aqueous flare at 1, 7, and 30 days according to surgical technique and degree of cataract nucleus hardness (using Pentacam Nucleus Staging Method).

Groups												
Parameters	Grade 1				Grade 2				Grade 3			
	Femto + rotation	Manual + rotation	Manual + chop	p	Femto + rotation	Manual + rotation	Manual + chop	p	Femto + rotation	Manual + rotation	Manual + chop	p
**Preoperative**												
CCT (mm)	549 ± 1	553 ± 3	550 ± 2	0.203	551 ± 9	555 ± 10	550 ± 6	0.326	549 ± 8	548 ± 8	547 ± 11	0.929
ECD (cells/mm^2)^	2831.15 ± 171.76	2672.63 ± 197.95	2940.73 ± 178.06	0.293	2704.29 ± 266.03	2627.66 ± 198.60	2699.11 ± 320.75	0.736	2497.33 ± 339.97	2491.96 ± 368.41	2513.48 ± 212.49	0.992
Aqueous flare(pc/ms)	6.30 ± 3.68	6.07 ± 0.31	6.83 ± 1.88	0.898	6.25 ± 3.36	6.39 ± 1.22	5.82 ± 1.42	0.793	5.55 ± 2.30	6.13 ± 1.26	7.17 ± 3.31	0.464
**Postoperative**												
**1 day**												
CDVA	0.06 ± 0.01	0.10 ± 0.02	0.11 ± 0.01	**<0.001**	0.11 ± 0.02	0.15 ± 0.02	0.18 ± 0.01	**<0.001**	0.13 ± 0.01	0.21 ± 0.01	0.27 ± 0.01	**<0.001**
CCT (mm)	557 ± 4	609 ± 1	620 ± 2	**<0.001**	581 ± 9	617 ± 5	622 ± 8	**<0.001**	620 ± 3	621 ± 4	635 ± 4	**<0.001**
ECL (%)	0.3 ± 0.2	2.8 ± 0.4	4.9 ± 0.6	**<0.001**	0.5 ± 0.2	3.4 ± 0.4	4.9 ± 0.4	**<0.001**	0.8 ± 0.1	5.0 ± 0.4	5.8 ± 0.9	**<0.001**
Aqueous flare(pc/ms)	11.30 ± 8.06	19.43 ± 1.69	21.97 ± 2.94	0.090	14.38 ± 2.74	18.74 ± 2.58	23.04 ± 4.86	**<0.001**	17.00 ± 3.68	19.69 ± 1.93	22.23 ± 5.26	0.059
**7 day**												
CDVA	0.01 ± 0.01	0.01 ± 0.01	0.05 ± 0.04	**<0.001**	0.01 ± 0.01	0.01 ± 0.01	0.08 ± 0.01	**<0.001**	0.01 ± 0.01	0.04 ± 0.01	0.10 ± 0.01	**<0.001**
CCT (mm)	550 ± 6	550 ± 7	552 ± 2	0.877	556 ± 7	557 ± 11	558 ± 4	0.859	575 ± 4	574 ± 3	575 ± 3	0.749
ECL (%)	3.4 ± 0.2	4.5 ± 0.3	5.1 ± 0.1	**0.001**	4.4 ± 0.4	5.7 ± 1.1	6.3 ± 0.4	**<0.001**	6.1 ± 0.5	7.2 ± 0.4	8.3 ± 0.5	**<0.001**
Aqueous flare(pc/ms)	10.35 ± 7.57	9.43 ± 2.31	15.77 ± 1.46	0.191	10.76 ± 3.45	11.59 ± 2.50	15.16 ± 2.84	**0.001**	8.24 ± 3.83	11.57 ± 2.05	13.58 ± 4.87	**0.043**
**30 day**												
CDVA	-0.12 ± 0.03	-0.12 ± 0.01	-0.12 ± 0.01	0.953	-0.01 ± 0.01	-0.12 ± 0.01	-0.12 ± 0.02	0.076	-0.11 ± 0.01	-0.11 ± 0.01	-0.12 ± 0.01	0.728
CCT (mm)	546 ± 1	548 ± 5	549 ± 1	0.532	552 ± 6	552 ± 4	554 ± 9	0.559	554 ± 6	554 ± 5	558 ± 1	0.222
ECL (%)	3.4 ± 0.1	6.3 ± 0.3	8.0 ± 0.2	**<0.001**	4.3 ± 0.5	8.8 ± 0.8	10.7 ± 0.9	**<0.001**	6.7 ± 0.3	10.2 ± 0.6	12.2 ± 0.9	**<0.001**
Aqueous flare(pc/ms)	6.85 ± 3.61	8.00 ± 1.35	9.50 ± 1.87	0.453	8.44 ± 3.21	8.73 ± 2.21	11.45 ± 3.40	**0.032**	7.13 ± 3.91	81.8 ± 2.25	11.25 ± 3.91	0.114

CCT = central corneal thickness; CDVA = corrected distance visual acuity (LogMAR); ECD = endothelial cell density; ECL = endothelial cell loss

Standard Snellen values converted into LogMAR units.

Anterior chamber aqueous flare was measured objectively using laser flare photometry (Kowa FM-600). Bold types indicate statistically significant difference among 3 subgroups in each group.
